# Adaptive vs Monthly Support for Weight-Loss Maintenance: A Randomized Clinical Trial

**DOI:** 10.1001/jamanetworkopen.2025.32681

**Published:** 2025-09-22

**Authors:** Kathryn M. Ross, Meena N. Shankar, Peihua Qiu, Zibo Tian, Jaime Ruiz, Lisa Anthony, Michael G. Perri

**Affiliations:** 1 Department of Clinical and Health Psychology, College of Public Health and Health Professions, University of Florida, Gainesville; 2 Advocate Aurora Research Institute, Advocate Health, Milwaukee, Wisconsin; 3 Department of Social Sciences and Health Policy, Division of Public Health Sciences, Wake Forest University School of Medicine, Winston-Salem, North Carolina; 4 Department of Biostatistics, College of Public Health and Health Professions and College of Medicine, University of Florida, Gainesville; 5 Department of Computer and Information Science and Engineering, Herbert Wertheim College of Engineering, University of Florida, Gainesville

## Abstract

**Importance:**

Weight regain is common after the end of initial weight-loss treatment. Existing extended care programs (which typically provide sessions once per month) improve long-term weight-loss outcomes, but with only modest effect vs control.

**Objective:**

To determine whether weight regain is reduced via provision of telephone-based extended care on an adaptive (triggered by a study algorithm that estimates when individuals are at high risk for weight regain) vs static (provided once per month) schedule.

**Design, Setting, and Participants:**

A randomized clinical trial conducted between October 2019 and November 2024 in areas within driving distance of Gainesville, Florida. Adults with obesity enrolled in a 16-week weight-loss program; those who lost 5% or more of their baseline weight were eligible for randomization to 1 of 2 maintenance care conditions.

**Intervention:**

Participants received 20 months of telephone-based extended care support, delivered individually by a trained interventionist, either on an adaptive or static schedule.

**Main Outcomes and Measures:**

The primary outcome was weight change from month 4 (end of initial intervention) to month 24.

**Results:**

The 255 participants (mean [SD] age, 50.6 [11.3] years; 209 [82.0%] women; 51 [20.0%] Black, 25 [9.8%] Hispanic or Latino, and 170 [66.7%] non-Hispanic White) were randomized to an adaptive (128 participants) or static (127 participants) group. Weight regain from month 4 to 24 was 1.27 (95% CI, 0.07 to 2.47) kg in the adaptive vs 1.75 (95% CI, 0.43 to 3.06) kg in the static group, with no difference by condition. At month 24, adaptive participants maintained a mean (SD) weight loss of 8.1% (7.8%) from initial intervention baseline while static participants maintained 7.9% (8.5%); there was not a significant difference in the proportion of participants maintaining weight loss of 5% or more (adaptive, 59.5%; static, 59.8%).

**Conclusions and relevance:**

In this randomized clinical trial, participants in both conditions were successful at maintaining initial weight loss 20 months after the end of a weight-loss program, but providing extended care on an adaptive schedule did not confer additional benefit vs the once-per-month static schedule. Future research should investigate whether more precise algorithms of high-risk periods for weight regain can be developed and whether these models can improve weight maintenance outcomes. Moreover, given success of participants in both conditions at maintaining initial weight loss, future research should also investigate methods of improving the implementation and dissemination potential of telephone-based extended care interventions.

**Trial Registration:**

ClinicalTrials.gov Identifier: NCT04116853

## Key Points

**Question:**

Does the provision of telephone-based extended care on an adaptive (triggered by a study algorithm identifying high risk for weight regain) vs static (provided once per month) schedule improve success at long-term weight-loss maintenance?

**Findings:**

Results of this randomized clinical trial with 255 participants demonstrated no difference in weight regain from month 4 to 24 in adaptive vs static groups. At month 24, 60% of participants across both groups maintained weight loss of 5% or greater from baseline.

**Meaning:**

These findings suggest provision of extended care on an adaptive vs static schedule did not reduce weight regain; however, the successful maintenance of clinically-meaningful weight loss in both conditions supports the role of extended care for weight-loss maintenance.

## Teaser

This randomized clinical trial evaluates whether weight regain is reduced via provision of telephone-based extended care on an adaptive schedule, triggered by a study algorithm that estimates when individuals are at high risk for weight regain, vs a static, once-per-month schedule.

## Introduction

Comprehensive weight-loss programs produce clinically meaningful weight loss in adults with obesity^1^; however, longer-term maintenance remains a challenge.^2^ Without additional intervention, most individuals regain weight after completion of initial weight-loss programs, regaining one-third to one-half of lost weight within the first year and gradually returning to baseline weight within 3 to 5 years.^3,4^ Extended care interventions that provide continued support in person or via telephone (typically as once-per-month sessions) have been the most effective method of promoting maintenance,^5,6,7^ but effects have been modest at best (resulting in approximately 2-3 kg less regain at 18-24 months^6,7^), suggesting the need for new approaches.

The proliferation of smartphones and digital-health tools have supported development of novel, adaptive intervention designs. For example, just-in-time adaptive interventions monitor individual factors (eg, moods, cognitions, and behaviors) to target intervention at key, high-risk periods.^8^ Our team previously developed an algorithm aimed at targeting extended care for when an individual is deemed to be at high risk for weight regain.^9^ Specifically, this algorithm uses data regarding individuals’ self-monitoring behaviors (frequency of self-monitoring dietary intake and weight) along with ratings of physiological states and cognitions (ratings of hunger and how important it was to stay on track with weight management goals given competing life priorities) in a given week to identify when an individual may be at risk for gaining weight the following week. We hypothesized that providing extended care intervention in a week that an individual is deemed to be at high risk for weight regain would be more adaptive to individual needs and improve long-term weight-loss outcomes.

The Support and Tracking to Achieve Results (Project STAR) trial evaluated the impact on weight regain of extended care provided on an adaptive schedule vs the static once-per-month schedule used in reference standard weight-loss maintenance programs. Adults with obesity who lost 5% or more of their baseline weight (deemed clinically meaningful by the Institute of Medicine^10^) during a 16-week weight-loss program were randomized to receive phone-based extended care support on either an adaptive or static schedule for an additional 20 months. Our primary aim was to test the hypothesis that an adaptive schedule would produce less weight regain than a static one from month 4 (end of initial intervention) to month 24. As a secondary aim, we tested the hypothesis that a greater proportion of adaptive participants would maintain clinically significant weight loss (of ≥5% from initial intervention baseline) at month 24.

## Methods

Project STAR was conducted across 5 cohorts between October 2019 and November 2024, using 2 phases: (1) a nonrandomized initial intervention phase (months 0-4) when all participants were provided with a 16-week lifestyle weight-loss program, and (2) a 20-month randomized trial phase (months 5-24) when eligible participants were randomized into 1 of 2 extended care maintenance programs. A full description of the study design, including enrollment and outcomes from the initial weight-loss program, has been published.^11^ Supplement 1 provides the full trial protocol. Study procedures were approved by the University of Florida institutional review board, and all participants provided written informed consent (during screening, before the weight-loss program). This trial followed the Consolidated Standards of Reporting Trials (CONSORT) reporting guideline.

### Participants

Participants were adults with obesity (aged 18-70 years, body mass index [BMI; calculated as weight in kilograms divided by height in meters squared] 30-45 with weight <180 kg due to study-provided e-scale limit) who reported owning a smartphone with a cellular data plan, living within driving distance of Gainesville, Florida, and no medical contraindications for weight loss. A mix of community-based recruitment methods (Supplement 1) were used to recruit participants for the initial intervention. The trial was powered at 0.90 to detect a mean (SD) difference in weight change of 2.5 (5.5) kg between adaptive and static groups from month 4 to 24 (2.5 kg was selected as the minimum threshold for clinical meaningfulness as weight changes of this magnitude have been demonstrated to reduce the risk of type 2 diabetes^1^). Assuming 60% of initial-intervention participants would be randomized to the maintenance trial and retention of 80% at month 24, we aimed to recruit 430 adults for the initial intervention to randomize 258 to the trial. We recruited 449 participants for the initial intervention and randomized 255.^11^

### Initial Weight-Loss Intervention

Participants received an initial 16-session weight-loss program based on the Diabetes Prevention Program, with weekly sessions led by trained interventionists with bachelor’s or master’s degrees in nutrition, psychology, exercise science, or a related field.^12^ The first study cohort began in person; however, sessions were transitioned to videoconference (via Zoom) starting with session 11 due to the COVID-19 pandemic. Intervention was delivered via videoconference for all remaining cohorts. Participants were provided with caloric intake goals (1200 to 1800 kcal/d, based on baseline weight) and physical activity goals (gradually increasing moderate-intensity physical activity, such as brisk walking, up to 300 minutes per week) designed to induce weight loss of approximately 0.45 to 0.91 kg per week.^1^ Participants were asked to self-monitor weight, dietary intake, and physical activity daily using study-provided tools (an e-scale [BodyTrace] and smartphone application [FatSecret]^13^).

### Randomization

The study statistician (P.Q.) randomized eligible participants to the parallel-group maintenance trial using 1:1 allocation, stratified by initial weight loss (ie, ≥10% or <10%) due to the known association between initial weight loss and long-term weight-loss maintenance.^14,15,16^ Randomization occurred following final determination of eligibility for the maintenance trial; the statistician had no access to participant data before randomization. The study team and analysts were not masked to condition after randomization.

### Maintenance Conditions

Throughout the maintenance trial, all participants were asked to continue to self-monitor weight, dietary intake, and physical activity daily and to download a smartphone application developed by our study team that synced with the study-provided e-scale and FatSecret application to display summaries of self-monitoring data (eg, total caloric intake vs caloric-intake goal, charts of caloric intake and physical activity over the prior week, and a graph demonstrating weight trajectory over time).^17^ This application also pushed notifications twice each week asking participants to complete brief self-report questionnaires. Every Sunday evening, participants were asked to complete 2 items asking them to rate (from 1-7) how hungry they were over the past week and how important it was for them to stay on track over the past week compared with other demands in their life. On another randomly-selected evening each week, participants were asked to complete a 12-item measure assessing other constructs hypothesized to be proximally associated with weight loss.^18^ Finally, all participants were provided with telephone-based intervention over the 20-month maintenance period, although the frequency and timing of sessions varied by condition. For each session, participants were contacted by their assigned study interventionist via telephone for a 10- to 20-minute call (up to 5 attempts made per call). No new session material was presented. Rather, calls followed a semistructured format that began with a brief check-in followed by a discussion of any barriers experienced by participants in meeting their weight maintenance goals; interventionists employed structured problem-solving techniques to help participants address these goals and goal-setting techniques to help them set new behavioral goals (see Supplement 1).^19,20^ Maintenance calls were made by the same interventionist throughout the trial (the same interventionist who led initial group sessions) whenever possible, with substitutions made as needed due to staffing changes.

#### Static Condition

Static participants were provided with up to 20 extended care calls on a fixed, once-per-month schedule, mirroring the frequency of contact used in reference standard extended care programs.^1,6^ The date and time of the initial call was scheduled shortly after notification of randomization and subsequent calls were scheduled at the end of each call.

#### Adaptive Condition

Adaptive participants were provided with extended care calls when either (1) our algorithm (published previously^11^) determined that a participant was at high risk for weight regain based on self-monitoring data and responses to Sunday questionnaire items (with missing data on rating scale questions also triggering an intervention call), or (2) the participant requested a session via the support section of the app (only visible to adaptive participants). Participants received 1 initial call to introduce their interventionist, discuss initial goals, and provide a list of days and times they would generally be available to receive calls. For subsequent calls, interventionists received an e-mail sent before the start of the workday each Monday listing which participants needed to be called. Interventionists then called these participants during the days and times they had indicated that they were available; participants who had a call triggered by our algorithm were not informed of the reason for the call (during the initial call, it was noted that the interventionist would call when our study app suggested a good time to check in).

### Measures

Weight was measured via e-scales that sent weights to research servers via the cellular network and have demonstrated high concordance with in-person assessment weights.^21,22^ Height was self-reported (both self-reported and staff-measured heights were available for cohort 1; concordance between these measures was high; Lin concordance, 0.98^11^). Race (American Indian or Alaskan Native, Asian, Black or African American, Native Hawaiian or Pacific Islander, White, and not reported) and ethnicity (Hispanic or Latino or not Hispanic or Latino) were self-reported on a demographics questionnaire, using categories defined by the National Institutes of Health and were assessed due to National Institutes of Health requirements. Call date and start and end time (along with number of attempts made for each call) were tracked by study interventionists.

### Statistical Analysis

Analyses used an intent-to-treat approach, including all randomized participants. A longitudinal mixed-effects model was used to test whether adaptive participants experienced less weight regain from month 4 to 24 compared with static participants, including fixed effects for randomization condition, time since study start, and initial intervention weight loss (a binary variable representing whether weight loss was ≥10%, used to stratify randomization). Although we initially proposed to use maximum likelihood estimation for missing data,^11^ the existence of missing data from participants who were withdrawn before both month 12 and 24 assessments precluded this approach; thus, multiple imputation (with 5 imputed datasets) was used. Sensitivity analyses were conducted using prespecified scenarios, including carrying-forward of the last daily e-scale weight and imputation of missing weights under the assumption (used in prior weight maintenance trials^23,24^) that participants regained 0.3 kg per month from their last e-scale weight. The pattern of results from these analyses were the same; thus results presented are from combining results across the 5 multiply-imputed datasets. Two-sided *t* tests were used to calculate *P* values, and significance was set at less than .05. Data were analyzed using T software version 4.3.1 (R Project for Statistical Computing).

## Results

### Participant Characteristics and Initial Intervention Outcomes

**Figure 1.**
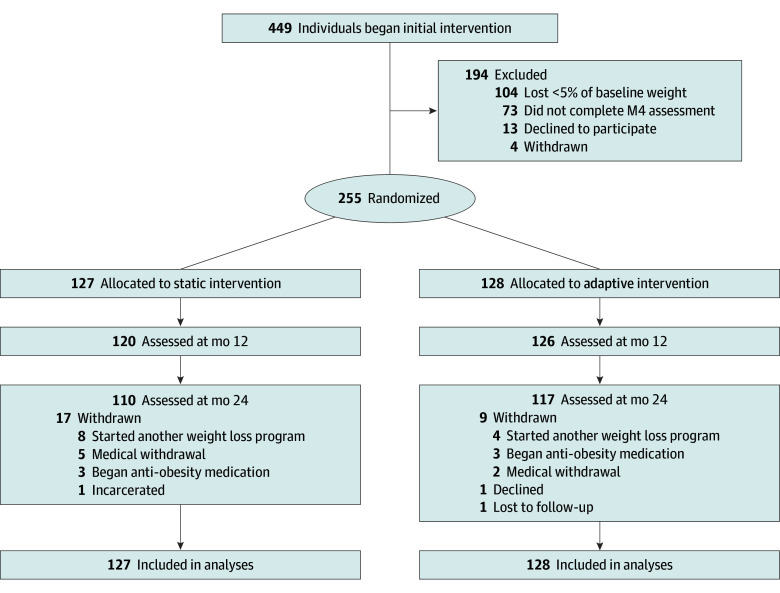
Participant Flow Diagram

A total of 255 participants were included in the Project STAR trial (Figure 1); 127 were randomized to the group static and 128 to the adaptive group. At initial intervention baseline, participants were aged a mean (SD) of 50.62 (11.30) years and had a BMI of 35.60 (4.15); 209 (82.0%) identified as women; 51 [20.0%] were Black, 25 [9.8%] were Hispanic or Latino, and 170 [66.7%] were non-Hispanic White. Table 1 provides participant characteristics by condition. Participants lost a mean (SD) of 9.51 (3.67) kg (9.53% [3.33%] of baseline weight) during the initial intervention, with no difference by condition.

**Table 1.** Participant Baseline and Demographic Characteristics

Characteristic	Participants, No. (%)	
	Static (n = 127)	Adaptive (n = 128)
Age, mean (SD), y	50.4 (12.1)	50.8 (10.5)
BMI, mean (SD)	35.5 (4.2)	35.7 (4.1)
Gender		
Man	28 (22.0)	18 (14.1)
Woman	99 (78.0)	110 (85.9)
Ethnicity		
Hispanic or Latino	12 (9.4)	13 (10.2)
Not Hispanic or Latino	115 (90.6)	115 (89.8)
Race^a^		
American Indian or Alaskan Native	2 (1.6)	2 (1.6)
Asian	5 (3.9)	2 (1.6)
Black or African American	30 (23.6)	21 (16.4)
Native Hawaiian or Pacific Islander	1 (0.8)	0
White	92 (72.4)	107 (83.6)
Not reported	2 (1.6)	1 (0.8)
Education		
High school	3 (2.4)	5 (3.9)
Vocational training or some college	14 (11.0)	20 (15.6)
Associate degree	14 (11.0)	13 (10.2)
College/university degree	44 (34.6)	36 (28.1)
Graduate or professional education	52 (40.9)	54 (42.2)
Marital status		
Married or living with significant other	85 (66.9)	96 (75.0)
Separated/divorced/widowed	20 (15.7)	20 (15.6)
Never married or other	22 (17.3)	12 (9.4)
Annual household income, $		
0-25 000	4 (3.1)	4 (3.1)
25 001-50 000	18 (14.2)	18 (14.1)
50 001-75 000	24 (18.9)	26 (20.3)
75 001-100 000	23 (18.1)	24 (18.8)
100 001-125 000	19 (15.0)	30 (23.4)
≥125 001	32 (25.2)	25 (19.5)
No response	7 (5.5)	1 (0.8)

Abbreviation: BMI, body mass index (calculated as weight in kilograms divided by height in meters squared).

^a^ Participants could select more than 1 race category; therefore, totals may exceed 100%.

### Retention and Maintenance Call Completion

Primary outcome data were available for 246 participants (96.5%) at month 12 and 227 (89.0%) at month 24, with no differences in retention by condition. Table 2 provides details on intervention call completion by condition. On average, static calls lasted longer than adaptive; however, as adaptive participants were scheduled for and completed more calls than static, their total call time was also higher (mean [SD] 6.4 [3.9] hours vs 4.8 [2.1] hours, respectively). Of the 7188 total adaptive calls attempted, 25 (0.35%) were triggered by 16 participants requesting support within the app; the remaining were triggered by the study algorithm. There was a decrease in the proportion of attempted calls completed in both groups between months 5 and 12 and 13 and 24; the adaptive group demonstrated a reduction from a mean (SD) of 69.9 (20.5%) to 47.1 (25.1%) calls; *t*_127_ = −14.1; *P* < .001; static from 98.8 (8.4%) to 95.2 (13.5%) calls; *t*_126_ = −3.55; *P* < .001. At month 24, 125 participants (63 adaptive and 62 static) completed a 5-point rating scale querying satisfaction with the number of calls (1 = not enough, 3 = just right, or 5 = too many); although a similar proportion of adaptive and static participants selected 3 (adaptive, 41 [65.1%]; static, 38 [61.3%]), more static participants selected 1 or 2 (adaptive; 9 [14.3%]; static; 19 [30.6%]) and more adaptive participants selected 4 or 5 (adaptive; 13 [20.6%]; static; 5 [8.1%]).

**Table 2.** Extended Care Intervention Call Completion by Condition

Variable	Mean (SD)		*P* value
	Static	Adaptive	
Call length, min	16.8 (5.1)	13.2 (4.3)	<.001
Total call time, min	288.0 (125.3)	383.5 (231.2)	<.001
Total calls completed, No.	17.2 (4.9)	29.5 (13.7)	<.001
Total calls attempted, No.	17.8 (4.6)	56.2 (20.4)	<.001
Proportion of calls completed, %	96.1 (9.9)	55.7 (21.4)	<.001

### Weight Change

**Figure 2.**
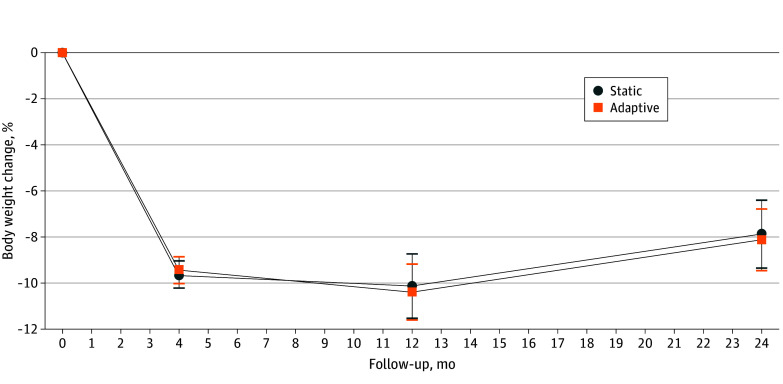
Model-Estimated Mean Change in Percentage Body Weight Over Time by Condition Error bars indicate 95% CIs.

Model-estimated weights at each assessment by condition are in Table 3. Figure 2 presents percentage weight change over time by condition. From month 4 to 24, adaptive participants regained a mean of 1.27 (95% CI, 0.07 to 2.47) kg while static participants regained a mean of 1.75 (95% CI, 0.43 to 3.06) kg, with no significant difference by condition. There was also not a significant difference between conditions in the proportion of participants maintaining weight loss of 5% or more from baseline at month 24 (adaptive, 59.5%; static, 59.8%). At month 24, adaptive participants maintained a mean (SD) weight loss of 8.1% (7.8%) from baseline whereas static participants maintained 7.9% (8.5%).

**Table 3.** Model-Estimated Weight by Assessment Time Point and Condition

Time	Participant weight, mean (SD), kg	
	Static	Adaptive
Baseline	100.22 (16.85)	99.57 (14.14)
Month 4	90.59 (15.75)	90.18 (13.28)
Month 12	90.15 (17.56)	89.18 (14.15)
Month 24	92.34 (17.71)	91.45 (14.92)

From month 4 to 12, adaptive participants lost an additional 1.00 (95% CI, −1.90 to −0.09) kg, whereas static participants demonstrated no significant change in weight (mean, −0.45; 95% CI, −1.57 to 0.67 kg). At month 12, 76.1% adaptive participants maintained losses of 5% or more from baseline vs 69.9% in the static group; however, this was not a statistically significant difference. From month 12 to 24, participants in both conditions experienced weight regain (2.27; 95% CI, 1.43 to 3.10 kg; and 2.19; 95% CI, 1.10 to 3.28 kg) in adaptive and static groups, respectively.

## Discussion

We conducted a randomized clinical trial to evaluate whether providing extended care on an adaptive (determined by a study algorithm that aimed to identify individuals at high risk for weight regain) vs static (once-per-month, as is common in reference standard interventions^6^) schedule produced less weight regain after initial weight loss in adults with obesity. Results demonstrated no significant differences in weight change between conditions from completion of an initial weight-loss program to the end of the 20-month trial. There was also not a significant difference between conditions in the proportion of participants maintaining clinically-meaningful weight loss of 5% or more from baseline at month 24, with 60% of participants in both conditions meeting this threshold.

The adaptive group continued to lose weight from month 4 through 12, whereas the static group did not experience a significant change in weight during this time, suggesting some early promise for the delivery of extended care on an adaptive schedule. Overall, however, adaptive participants demonstrated only approximately 0.5 kg less weight regain than static participants at month 24, which was below our prespecified metric for assessing clinically meaningful difference (2.5 kg), suggesting no additional longer-term benefit for adaptive over static*, *perhaps due to the sharp decrease in call completion observed in adaptive (from 70% in months 5-12 to 47% in months 13-24). Given the association between session attendance and success with weight loss and maintenance,^25,26,27^ the diminished benefit of the adaptive schedule at month 24 may have been impacted by low call completion. A clinical observation from our interventionists was that the large number of call attempts in the adaptive group may have overburdened participants (indeed, more adaptive participants endorsed having too many calls). As our algorithm was optimized for sensitivity over specificity,^9^ participants may have received more calls than were necessary; our team intends to conduct additional investigation into the algorithm results, developing a modified algorithm with improved specificity (reducing the number of unnecessary calls). Future studies should also investigate whether providing extended care support via alternative or less-burdensome modalities (eg, text message, smartphone application prompt, or email), especially in a triaged manner (eg, reserving phone contact for participants experiencing larger challenges), can improve engagement with extended care delivered on an adaptive schedule and whether allowing participants to select the dose of extended care contact may be beneficial (eg, as observed in a previous text-based extended care intervention^28^).

Collectively, results also highlight the overall importance of extended care provision, supporting calls for obesity to be treated under a continuous-care model.^29^ Impressively, adaptive and static participants maintained 87% and 82% of their initial weight loss at month 24, respectively. Both conditions demonstrated less weight regain than observed in previous maintenance trials (with regain of 1.3 kg and 1.8 kg for adaptive and static, respectively, at 20 months after initial intervention); after 18 months, participants receiving face-to-face extended care intervention in the STOP Regain trial regained 2.5 kg^24^ and participants receiving individual phone-based extended care (similar to the design of our static condition) in the Rural Lifestyle Eating and Activity Program (LEAP) trial regained 2.3 kg.^23^ Although the current trial did not have a no or minimal contact control condition due to equipoise, these previous trials demonstrated weight regains of 4.9 kg and 4.1 kg for control participants receiving newsletters or mailed educational materials, respectively.^23,24^ The tendency for the static group to demonstrate superior weight maintenance vs historic trial outcomes may be related to the additional technology integration^30^ in our static condition or the high completion of sessions by static participants (96%); attendance in the face-to-face STOP Regain condition was 42% to 79% over the maintenance period^24^ and the individual condition in Rural LEAP had a mean of 67% of calls completed.^23^ This high level of contact may have been sufficient to reach a maintenance asymptote that could not be exceeded with the additional support provided in the adaptive condition.

### Strengths and Limitations

Strengths of this study included implementation of a 2-phase design (used in previous maintenance trials^24^) wherein only individuals who lost weight were enrolled in the maintenance trial, isolating maintenance intervention effects. There was also minimal attrition; missing data were largely related to withdrawal and few participants were lost to follow-up. Despite these strengths, generalizability of results to the broader population of adults with obesity may be limited. Our sample was predominately female and reported high levels of education and income, and although 33% of participants were from racial and ethnic backgrounds historically underrepresented in research (including 20% who identified as African American), Hispanic adults were underrepresented in our trial (10% of our sample). Our algorithm was also not tailored by gender, education, income, or race and ethnicity, which may have limited its effectiveness for triggering interventions; future studies should investigate whether intervention provision (and/or content) should be tailored by individual demographic factors, allowing for precision health approaches to obesity treatment. Additionally, the trial did not include a no or minimal contact control group; all participants in the current study received extended care support, limiting comparison of outcomes.

## Conclusions

Although there was some evidence for early benefit of providing phone-based extended care on an adaptive schedule, there was not a significant difference in weight regain between adaptive and static groups at month 24. Overall, the success of both adaptive and static interventions at promoting weight-loss maintenance highlights the importance of providing extended care interventions under the continuous-care model of obesity treatment. Future research should investigate whether more precise algorithms to estimate high risk periods for weight regain can be developed and whether these models improve weight maintenance outcomes.

## Notes

Supplement 1.
Trial Protocol

Supplement 2.
Data Sharing Statement
