# Supplementary material for: Adaptive vs Monthly Support for Weight-Loss Maintenance: A Randomized Clinical Trial
Source: JAMA Netw Open. 2025 Sep 22;8(9):e2532681. doi: 10.1001/jamanetworkopen.2025.32681 (PMC12455375; doi:10.1001/jamanetworkopen.2025.32681)
Supplement: Supplement 1. — Trial Protocol [file jamanetwopen-e2532681-s001.pdf]

EVALUATION OF AN ADAPTIVE INTERVENTION FOR WEIGHT LOSS MAINTENANCE

Protocol Number: IRB201803061
National Clinical Trial (NCT) Identified Number: NCT04116853

Principal Investigator: Kathryn M. Ross, PhD, MPH

Sponsor: University of Florida

Grant Title: Evaluation of an Adaptive Intervention for Weight Loss Maintenance

Grant Number: 1 R01 DK119244-01A1

Funded by: The National Institute of Diabetes and Digestive and Kidney Diseases
6/17/2024

1. **Project Title:**

   Evaluation of an Adaptive Intervention for Weight Loss Maintenance

2. **Investigators:**

   Principal Investigator:

   Kathryn M. Ross, Ph.D., M.P.H.
   Associate Professor
   Department of Clinical and Health Psychology
   College of Public Health and Health Professions
   University of Florida
   P.O. Box 100165
   (352) 294-8433

   Co-Investigator(s):

   Lisa Anthony, Ph.D.
   Associate Professor
   Department of Computer and Information Science and Engineering
   Herbert Wertheim College of Engineering
   University of Florida
   P.O. Box 116120
   (352) 392-1200

   Jamie Ruiz, Ph.D.
   Associate Professor
   Department of Computer and Information Science and Engineering
   Herbert Wertheim College of Engineering
   University of Florida
   P.O. Box 116120
   (352) 392-1200

   Peihua Qiu, Ph.D.
   Professor
   Department of Biostatistics
   College of Public Health and Health Professions
   University of Florida
   P.O. Box 117450
   (352) 294-5911

3. **Abstract:**

   Participant engagement/attendance in existing extended-care interventions remains suboptimal, limiting effectiveness for long-term weight loss maintenance. We propose to evaluate a method for providing phone-based extended-care intervention *adapted to participant needs* (e.g., when participants are at "high risk" for weight regain as assessed by a predictive algorithm). We propose to conduct a randomized controlled trial assessing the impact of phone-based extended care delivered on an *ADAPTIVE* (only when indicated via our algorithm or when requested in-app by participants) vs. *STATIC* (pre-scheduled, once-per-month frequency used in existing extended-care programs) schedule on weight loss maintenance. As we propose to study weight loss *maintenance*, a key design consideration was to only randomize participants who have been successful with initial weight loss.[1] Similar to existing maintenance trials,[2,3] we will provide participants with an initial group-based gold-standard behavioral weight management program and then randomize participants who successfully achieve a clinically-significant weight loss between baseline and Month 4 (defined by the Institute of Medicine[4] as a reduction in weight of ≥ 5% from baseline).

4. Background:

Obesity remains a substantial public health challenge in the United States. While behavioral lifestyle interventions have been demonstrated to produce weight losses of 8-10% in adults with overweight and obesity,[5] long-term outcomes are suboptimal.[1] Following the end of initial treatment, individuals typically regain 1/3 to 1/2 of lost weight within a year and gradually return to baseline weight in 3-5 years.[6] Research has demonstrated, however, that individuals who are able to continue to adhere to weight management behaviors can successfully maintain their weight loss long-term,[5,7] implicating non-adherence to the eating and activity changes that produce weight loss as the primary mechanism for this weight regain.[1,8,9]

As a result, current clinical guidelines now conceptualize obesity within a chronic disease "continual care" model, necessitating long-term treatment provision.[2,8,10,11] Existing "extended-care" programs typically provide long-term support via monthly sessions (delivered in-person or via telephone) and have demonstrated significant improvements in adherence to behavior change and less weight regain compared to no-contact control.[12] While these effects have been *statistically significant*, the impact of existing programs on weight regain has been *modest* (around 1.5 kg less regain vs. control at 24 months)[13] and there tends to be high individual variability in response to treatment.[1]

A key challenge to the effectiveness of extended-care programs has been continued participant engagement, typically operationalized as session attendance. Attendance has been consistently associated with long-term weight loss maintenance;[3,12,14] however, attendance at monthly extended-care sessions tends to be poor and declines over time.[6] The once-per-month, static treatment schedules of existing programs may contribute to these suboptimal outcomes.[15] Under this schedule, a participant experiencing a small lapse (e.g., failing to self-monitor dietary intake for several days) may not receive additional support for several weeks, by which time they may be experiencing a larger lapse (e.g., complete abandonment of self-monitoring and former dietary changes) and weight regain. Importantly, this cycle may result in participant disengagement and avoidance of treatment sessions due to feelings of frustration, shame, or embarrassment.[16–18] In contrast, tailoring intervention delivery such that extended-care sessions are provided when individuals are at "high risk" for weight regain offers potential to disrupt this cycle and significantly improve program engagement, adherence to program goals, and long-term weight maintenance outcomes.

Dr. Ross is currently PI of a NIDDK-funded grant (R21DK109205) aimed at identifying and characterizing "high risk" periods of weight regain. For this project, Dr. Ross and Dr. Qiu (Co-I) first developed longitudinal change point mixture models[19] to model the trajectory of weight loss and regain in 75 adults with overweight and obesity who took part in a 12-week Internet-based weight management program[20] followed by a 9-month maintenance observation period during which no additional intervention contact was provided. Next, longitudinal multilevel models were used to identify factors proximally (on a week-to-week basis) associated with weight regain after initial weight loss. During the full study year (including the 9-month maintenance period), participants used a study website each week to self-report the number of days they monitored their weight, dietary intake, and total minutes of physical activity and completed an 11-item questionnaire asking them to rate, on 7-point Likert scales, factors that had been hypothesized previously to be associated with weight regain (e.g., positive mood, negative mood, stress, hunger, boredom with weight control efforts, temptation to eat foods not on their plan, temptation to skip planned physical activity, the degree to which physical activity choices were consistent with weight loss goals, the amount of effort that it took to stay on track, and the importance of staying on track, compared to other demands in life during the previous week). In a sub-sample of participants from the initial dataset who had successfully lost ≥ 5% of their baseline weight during the initial 12-week intervention (n=46), these results were used to fit regression tree models.[21,22] These models were developed using R[23] package *rpart*,[24] using trimming methods proposed by Brieman,[21] and predicted weight change the following week as a continuous response variable.

Drs. Ross and Qiu evaluated the optimized model in a test dataset consisting of the remaining 28 study participants who continued to submit data during the 9-month maintenance period but whose data were not used to develop the predictive algorithm. In this separate test dataset, the

algorithm demonstrated a sensitivity of 82.0% and a specificity of 30.4%. Taken together, these promising results suggested two key implications: 1) that weight regain can be reliably modeled and predicted, and 2) that this optimized predictive algorithm demonstrated good performance both in the initial training and separate testing datasets.

In the current study, we propose to evaluate an innovative method of providing phone-based extended-care treatment *adaptive to participant needs.* The proliferation of smartphones and mHealth apps has allowed for the development of new, adaptive treatment models aimed at providing intervention when it is needed most.[25] Monitoring early signals of weight regain via an mHealth app will allow us to tailor intervention delivery at a previously-impossible level, via a method that is convenient and acceptable to participants. The dataset resulting from the current trial will be unique in terms of both *scope* and *resolution*, allowing us to build innovative behavioral models to inform future just-in-time adaptive intervention development.

5. **Specific Aims:**

Aim 1: Test the hypothesis that *ADAPTIVE* participants will experience significantly less weight regain than *STATIC* participants between Month 4 (randomization) and Month 24.

Aim 2: Test the hypothesis that, compared to *STATIC*, a significantly greater proportion of *ADAPTIVE* participants will maintain clinically significant (≥ 5% from baseline[4]) weight losses at Month 24.

Exploratory Aims: We propose to 1) use daily weight data collected via our mHealth app to model differences in weight trajectories between groups; 2) investigate potential treatment mediators (e.g., treatment engagement/session attendance and adherence to diet, physical activity, and self-monitoring goals); 3) use the rich dataset collected via our mHealth app to develop dynamic models of weight loss/regain and to investigate additional proximal predictors of weight-related behaviors, identifying potential decision points and tailoring variables for future just-in-time adaptive intervention development.

6. **Research Plan:**

Inclusion Criteria

- Age 18-70
- BMI 30.0-45.0 kg/m^2
- ≤ 396 pounds (due to scale limit)
- Own a smartphone [Apple iPhone (5s or newer) or Android smartphone (KitKat OS 4.4 or newer)] with a cellular and data plan
- Lose ≥ 5% of baseline weight during the initial weight loss intervention (Month 0 to Month 4)
- Willing to complete a phone-based maintenance program
- Completion of baseline assessment measures

Exclusion Criteria

- History of bariatric surgery
- Current use of weight loss medications
- Weight loss of ≥ 10 lb in 6 months prior to screening for initial intervention enrollment
- Physical limitations that prevent walking ¼ mile without stopping
- Currently pregnant
- Currently breastfeeding
- Less than 1 year post-partum
- Plans to become pregnant within the study period
- Lack of written approval for participation from potential participant's physician if the participant has been diagnosed with type 2 diabetes, hypertension, or has a history of coronary heart disease
- More than one participant per household (enrollment limited to one participant per household)
- Medical conditions that contraindicate weight loss or prevent completion of the study (e.g., current diagnosis of cancer or terminal illness, dementia, etc.)

- Unable to complete the two-year study (due to plans to relocate during the study period, etc.)
- Unwilling or unable to provide informed consent
- Unable to read English at the 5th grade level
- Unwilling to accept random assignment
- Unwilling to use the study app
- Unwilling to travel to initial intervention sessions
- Participation in another randomized research project
- Other conditions which in the opinion of the Principal Investigator would adversely affect participation in the trial

Recruitment and Screening

Recruitment. Based on experience in previous trials, we anticipate needing to screen approximately 1230 people to obtain a sample of 430. Participants will be recruited using 1) flyers, 2) in-person lectures and events, 3) contacting individuals who agreed to be recontacted for future research by signing a Consent2ShareThe Consent2Share process is a part of the University of Florida (UF) Clinical and Translational Science Institute's (CTSI) Integrated Data Repository, a database organizing clinical information across UF Health's clinical and research programs[26], 4) outreach to local employers/businesses with IRB-approved materials, 5) newspaper, newsletter, print, and radio ads, 6) UF Health Studies website, 7) Direct mailings using publicly available mailing lists, 8) IRB-approved flyer shared via Nextdoor, a closed neighborhood-based social media site that allows neighbors to share important information, alerts, and updates with fellow community members,9) ResearchMatch.org, a national electronic, web-based recruitment tool that was created through the Clinical and Translational Science Awards Consortium, and 10) Facebook posts via local businesses, organizations, and churches using IRB-approved materials.

Phone Screens. Potential participants who call or email us for more information will receive a description of the study and will be asked to complete a brief phone screen questionnaire assessing for basic eligibility criteria (e.g., age, BMI, history of bariatric surgery, medical conditions that would contraindicate weight loss). Potentially eligible participants will be asked to attend an orientation visit in order to learn more detailed information about the study and complete final measures to determine if they are eligible to participate. Participants will be asked to select from a range of days/times to attend this orientation visit, and will be emailed confirmation of their selected day/time.

Orientation and final eligibility screening. Participants who meet initial eligibility criteria as assessed via the phone screen will be invited to attend an orientation visit. These visits will be conducted in small groups, and will begin with a discussion of the pros/cons of taking part in research and then discuss specific details of the current study (including but not limited to intervention protocol, the number and timing of follow-up assessment visits, and risks and benefits of taking part in the trial). If social distancing procedures are in effect, the orientation visit will take place by Zoom UFLPHI with e-consenting via REDCap. Potential participants will be given the opportunity to privately ask study staff any remaining questions that they have and then, if they remain interested, will be asked to provide written informed consent.

Screening Visit. After signing written informed consent, participants will complete baseline screening measures (see Table 1). If social distancing procedures are in effect, a study-provided scale will be mailed to participants for weight to be measured. At this time, participants will be asked to indicate what days/times they are available to attend intervention groups. Participants will be contacted via phone and informed of their final eligibility. During this call, participants who remain eligible for the initial weight loss program will be informed of their group day/time.

Baseline (Month 0) Assessment Visit. The Month 0 assessment visit will take place before the lifestyle weight management program. Once participants have completed baseline measures, they will begin the initial weight loss program.

Eligibility for the Maintenance Program. Eligibility for the randomized controlled trial of the *ADAPTIVE* versus *STATIC* maintenance program will be determined after the Month 4 assessment visit. Percent weight change from the Month 0 assessment to the Month 4 assessment will be calculated; participants who lose ≥ 5% of their Month 0 weight at Month 4 will remain eligible for the maintenance trial.

Intervention and Randomization

Initial Weight Loss Program. All participants will be asked to complete a gold-standard 16-week lifestyle weight management program.[10] Similar to existing protocols, this initial intervention will be delivered via in-person group classes of 10-20 participants, led by trained Group Leaders with a bachelor's or master's degree in nutrition, psychology, exercise science, or a related field.[3,5,27] In the event of University closures, emergencies, or while social distancing procedures are in effect, this initial intervention will be delivered by Zoom UFLPHI. Zoom UFLPHI does not have access to identifiable health information and will protect and encrypt all audio, video, and screen sharing data. Meeting entry will require passwords which will be sent to participants separately from the meeting link. Intervention content for this 16-week program has been adapted from materials used in the 16-session initial treatment program in Rural LITE[28] and the 16-session "core curriculum" of the Diabetes Prevention Program.[29] Groups will meet weekly for 16 weeks.

Throughout the 16-week program, participants will be encouraged to follow a nutritionally-balanced reduced calorie diet (ranging from 1,200 to 1,800 kcal/day[10] based on starting weight). Further, participants will be encouraged to gradually increase engagement in physical activity to reach ultimate program goals of 10,000 steps per day[30] and 200-300 minutes per week of moderate-intensity activity[10,31] such as brisk walking. Consistent with current clinical guidelines for the treatment of obesity,[10] these eating and activity goals were designed to result in a weight loss of 1-2 lbs per week.

Participants will be asked to self-monitor weight daily, using a study-provided BodyTrace scale. Participants will be asked to self-monitor caloric intake and physical activity via the commercial FatSecret mobile app (provided free of charge to participants). This application can be accessed by participants via web (https://www.fatsecret.com/) or via smartphone application. At the first study intervention session, participants will be asked to download this application on their mobile devices and will be guided on how to set up accounts using either their preferred email address or a study-generated email address with no direct link to identifiable information. Participants will then be shown how to use this application to record their body weight, physical activity, and caloric intake each day. Participants will also be given guidance on how to protect their privacy and confidentiality using study devices and smartphone applications (e.g., suggesting using device encryption, using secure passwords and setting up automatic lock screens, and avoiding submitting personally-identifiable information through the applications).

Randomization. Participants who successfully achieve weight losses of ≥ 5% of initial body weight during the initial 16-week weight loss intervention (defined as change in assessment-measured body weight from Month 0 to Month 4) will be eligible for randomization for the maintenance program. After determining eligibility, participants will be randomized using a 1:1 allocation scheme by the study statistician (Dr. Qiu), stratified by initial weight loss (due to the known association between initial weight loss and longer-term maintenance,[32,33] participants will be categorized by whether initial weight loss was ≥10% or < 10%). Participants will be notified of their inclusion/exclusion and asked to return for one in-person study visit to be informed of their randomization (see full description below). This process will take place by phone if social distancing procedures are in effect.

Extended-Care Maintenance Intervention. All participants who are randomized at Month 4 will be asked to attend a single in-person study visit where they are informed of their study randomization, asked to provide general availability for extended-care maintenance intervention phone calls, and asked to download the MyTrack+ smartphone application developed by our study team and will be taught how to use this application. At this time, participants will again be given guidance on how to protect their privacy and confidentiality using study devices and smartphone applications (e.g., suggesting using device encryption, using secure passwords and setting up automatic lock screens, and avoiding submitting personally-identifiable information through the applications). Participants will be asked to use the MyTrack+ application to launch FatSecret, allowing them to continue to self-monitor their weight, physical activity, and caloric intake each day. If social distancing procedures are in effect, this visit will take place by phone.

MyTrack+ will link with the FatSecret application through an API link, using a de-identified study ID to link participant self-monitoring data and display this data within the MyTrack+ application. Further, MyTrack+ will send prompts to study participants asking them to complete brief questionnaires (see full description in the measures section). All randomized participants will receive extended-care intervention delivered via phone; however, the frequency/timing of these calls will vary between the *ADAPTIVE* and *STATIC* conditions. Similar to protocols for phone-based intervention sessions previous trials conducted by the PI[34] and the ongoing Rural LEAP trial, each phone-based extended-care session will last for approximately 10-20 minutes. Each call will be initiated by the interventionist, and will begin with a brief check-in followed by a discussion of any barriers experienced by participants in meeting their weight maintenance goals. To address these barriers, interventionists will guide participants through a five-step problem solving model,[35] including 1) positive problem orientation (developing a positive mindset), 2) problem definition (the concrete definition of the problem; i.e., the *who*, *what*, *when*, *where*, and *why* of the problem), 3) generation of alternatives (a brainstorming session where the goal is to produce the maximum number of potential solutions), 4) decision making (the evaluation of potential solutions by two key criteria: "*will this work if I do it?"* and *"what are the odds I'd actually do it?"*) and 5) solution implementation and verification (i.e., trying out the proposed solution and evaluating effectiveness). Each call will end with a formal goal setting session wherein participants are encouraged to set SMART (Specific, Measurable, Achievable, Relevant, and Time-limited) goals.[36–39] Interventionists will keep notes on each session so that he/she can check in with each participant during the next call regarding success toward stated goals (and success of any problem solutions identified). Further, the start and end time of each call will be documented to monitor the frequency/duration of intervention calls, and each call will be audio recorded to monitor intervention fidelity.

If the participant cannot be reached for a call, the interventionist will leave a voicemail asking the participant to return his/her call. Interventionists will make up to 5 "call attempts" on various days/times (ideally no more than one call per day) if unsuccessful. If the participant cannot be contacted after 5 attempts, the interventionist will attempt to contact the individual via email to note difficulty reaching the participant. If the interventionist does not receive a response to the email within a week, he/she will send a certified letter to the participant's home address noting the difficulty contacting them and to encourage continued participation in the study.

***STATIC* Extended-Care Program.** Participants randomized to the *STATIC* maintenance program will receive the extended-care intervention phone calls described above on a fixed, once-per-month schedule. The initial call date/time will be scheduled during each participant's randomization notification call. At the end of the initial phone session (and each session thereafter), interventionists will confirm the next call's scheduled day/time (one month after the current call, at the same time) and reschedule if necessary (e.g., participant time conflicts due to work or travel).

***ADAPTIVE* Extended-Care Program.** For participants randomized to the *ADAPTIVE* maintenance program, the extended-care intervention phone calls described above will be delivered only if either 1) an algorithm developed by our study team detects that a participant is at "high risk" for weight regain[40] or 2) the participant self-initiates a request for a session by navigating to a "support" section of the app. The algorithm that will be used to trigger intervention is provided in Figure 1;[41] the collection of data required for this algorithm will be conducted via MyTrack+ and is described in detail in the measures section.

Once intervention has been triggered (via our algorithm or an in-app support request), a notification will be automatically sent to the participant's interventionist via a secure email, and the interventionist will initiate an intervention call as soon as possible (i.e., at the next day/time the participant indicated they were generally available for intervention calls). On weeks that intervention is not triggered via our algorithm or via in-app participant request, no additional intervention contact will be provided

Interventionist Training. Interventionists will have a bachelor's or master's degree in nutrition, psychology, exercise science, or a related field. All interventionists will undergo training and certification in the intervention methods and treatment protocols prior to the start of the weight loss intervention. Interventionists will be provided with a comprehensive treatment manual (detailing session-by-session objectives and methods) and will be asked to attend a two-day workshop prior to the start of intervention. Prior to the end of the initial (in-person, group-based) weight management groups, all interventionists will be asked to attend a follow-up workshop focused on procedures for the *ADAPTIVE* and *STATIC* conditions and protocol for the phone-based extended-care sessions.

Figure 1. Final *ADAPTIVE* Treatment Delivery Algorithm

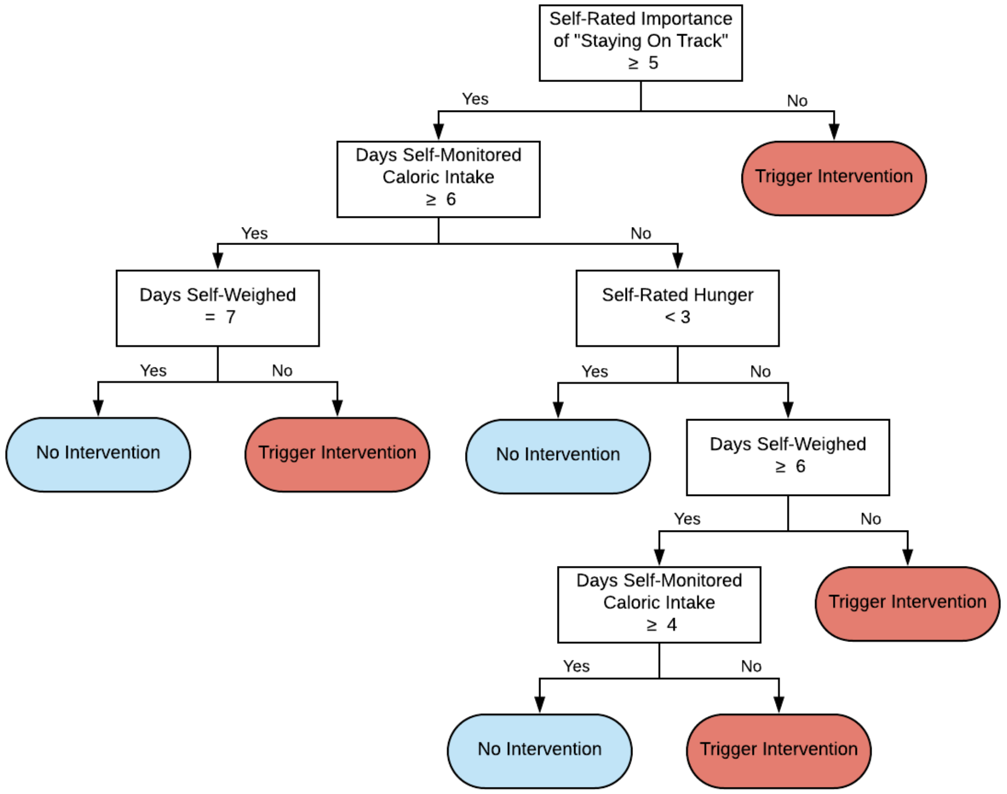

Weekly intervention team meetings led by the PI and the Research Coordinator will be dedicated to review of intervention materials, practice in intervention application (e.g., role play and rehearsal activities), and case management reviews of individual participant progress. All intervention sessions (both initial, in-person group sessions and extended-care phone sessions) will be audio recorded and random reviews will be conducted by to monitor fidelity and assess whether interventionists require additional training. At the end of the trial, 10% of all extended-care phone calls will be randomly selected and assessed for fidelity (using a fidelity checklist) by study staff.

Assessment Schedule and Measures

Assessments will be conducted at Month 0 (baseline), Month 4 (end of the initial weight loss intervention), Month 12, and Month 24 (see **Table 1**). Assessments will be conducted by trained study staff members masked to maintenance condition. All self-report questionnaires will be completed online via the University of Florida's REDCap (Research Electronic Data Capture)[42] system.

<u>Height</u>. Trained study staff will measure participant height via stadiometer, with participant shoes removed. If social distancing procedures are in effect, a self-reported height will be recorded.

<u>Assessment weight</u>. Participant body weight will be measured at the screening visit as well as all assessment visits by trained research staff using calibrated Tanita digital scales, with participants in light indoor clothing, with pockets emptied and belts and shoes removed. If social distancing procedures are in effect, weight will be measured using a study-provided BodyTrace smart scale.

<u>Demographics</u>. Participant demographic information will be collected via a brief demographics questionnaire completed at the screening visit.

<u>Contact Information</u>. Participant contact information will be collected via questionnaire at the screening visit. Contact information will be updated with any changes during the Month 4 and

12 visits.

Table 1. Schedule of Assessment Visits and Measures					
	Assessment Time Point				
		Initial Intervention		Extended-Care	
**Measure**	Screening Visit	Month 0	Month 4	Month 12	Month 24
**Anthropometrics**					
Weight (via Tanita digital scale)	x	x	x	x	x
Height (via stadiometer)	x				
**Questionnaires**					
Demographics	x				
Contact Information	x		x	x	
Weight Loss History		x			
WEL-SF (Weight Loss Self-Efficacy)[44]		x	x	x	x
WCSS (Weight Control Strategies)[45]		x	x	x	x
Medical History/Update	x		x	x	x
ASA24 Dietary Recall[43]		x	x	x	x
Cost Analysis Survey		x	x	x	x
Sedentary Behavior Questionnaire[46]		x	x	x	x
Munich Chronotype Questionnaire[47]		x			
Social Norms Scale[48]		x			
International Physical Activity Questionnaire[49]		x	x	x	x
Supportive Accountability Questionnaire[50]		x	x	x	x
Perceived Stress Scale[51]		x	x	x	x
Palatable Eating Motives Scale[52]		x	x	x	x
Meal Planning Questionnaire		x	x	x	x
EQ-5D-5L[53]		x	x	x	x
OPTION Scale[54]		x	x	x	x
Body Image States Scale[55]		x	x	x	x
Self-Compassion Scale[54]		x	x	x	x
Program Satisfaction Questionnaire					x
**Adherence/Other**					
Physical Activity (via ActiGraph)[55]		x	x	x	x
Session attendance		*Assessed Continuously*			
Weight (via in-home BodyTrace scale)		*Assessed Continuously*			
Self-monitoring (weight, dietary intake, activity)		*Assessed Continuously*			
Questionnaire data from MyTrack+		*Assessed Continuously*			

Weight Loss History. Weight loss history will be assessed via an 8-item self-report questionnaire completed at the baseline assessment visit.

Weight Loss Self-Efficacy. Self-efficacy for weight loss will be assessed via the Weight Loss Self-Efficacy Scale, an 8-item self-report questionnaire completed at all assessment time points.

Weight Control Strategies. Use of common weight control strategies will be assessed via the Weight Control Strategies Scale, a 30-item self-report questionnaire completed at all assessment time points.

Medical History/Update. Medical history will be collected at baseline via a self-report questionnaire. Updates to this history will be collected via a self-report questionnaire delivered at all remaining assessment visits.

Dietary Intake. Dietary intake will be assessed at all assessment time points using Automated Self-Administered 24-hour dietary recalls (ASA24)[43] completed by participants via the National Cancer Institute (NCI) respondent website. At each assessment time point, participants will be asked to complete three ASA24 recalls. Participants will be asked to complete these recalls on a schedule that is unknown to them; they will be informed that they will be contacted by study staff via email on the day that a recall is scheduled to be completed. At this point, participants will be asked to log on to the NCI ASA24 respondent website and to report all foods/drinks consumed on the prior day. Recalls will be scheduled by study staff so that each participant completes at least 2 recalls reflecting weekday intake and 1 recall reflecting weekend intake during each assessment time point.

Cost Analysis Survey. Healthcare costs will be assessed at all assessment time points using a 21-item self-report survey.

Sedentary Behavior Questionnaire. Weekday and weekend sedentary behavior will be assessed at all assessment time points using a 16-item self-report questionnaire.

Munich Chronotype Questionnaire. Sleep duration and timing will be assessed at Month 0 using a 30-item self-report questionnaire.

Social Norms Scale. Social norms for unhealthy eating and physical inactivity will be assessed at Month 0 using a 28-item self-report measure.

International Physical Activity Questionnaire. The long format of this questionnaire is a 27-item self-report survey which will be used to assess physical activity at all assessment time points.

Supportive Accountability Questionnaire. Social support and supportive accountability will be assessed at all assessment time points using a 33-item self-report measure.

Perceived Stress Scale. Perception of stress will be assessed at all assessment time points using a 10-item self-report measure.

Palatable Eating Motives Scale. Reasons for eating palatable foods outside of hunger will be assessed at all assessment time points using a 20-item self-report measure.

Meal Planning Questionnaire (MPQ). The MPQ is a new self-report questionnaire that has been developed by our study team in order to assess frequency with which individuals plan meals in advance. The MPQ will be administered at all assessment time points.

EQ-5D-5L. The EQ-5D-5L is a 6-item self-report measure of health-related quality of life which will be administered at all assessment time points.

OPTION Scale. The OPTION scale is a 10-item self-report measure of diet, physical activity, and sleep for parents of children under 18 years old which will be administered at all assessment time points.

Body Image States Scale (BISS). The Body Images States scale is a 6-item self-report measure of body image evaluation.

Physical Activity.

Physical activity will be assessed at all assessment time points using a hip-worn ActiGraph physical activity monitor. Participants will be given instructions to wear the monitor for seven days after each assessment time point.

Self-Compassion Scale.

The Self-Compassion Scale is a 26-item self-report measure of trait levels of self-compassion which will be administered at all time points.

Program Satisfaction Questionnaire. The program satisfaction questionnaire is a 16-item self-report questionnaire which will be administered at Month 24 to obtain participant feedback about the study.

Data collected through the study.

Audio Recordings of Intervention Sessions.

Audio recordings of intervention sessions will be collected for training and supervision purposes and to monitor intervention fidelity. Audio recordings will be transcribed and de-identified for purposes of coding nutrition/activity related issues and interventions. Audio recordings will be collected via Sony Digital Voice Recorders (phone sessions will be recorded by connecting these recorders to a phone adapter). These recorders will be kept in locked filing cabinets within the PI's locked research lab when not in use. Recordings will be downloaded to secure drives on the UF network (the Public Health and Health Professions P: drive under a secure folder only accessible to interventionists, the PI, and the study Research Coordinator) after each intervention session and deleted from the Sony Digital Voice Recorder.

Smart Scale Data. Throughout the initial 16-week intervention and until the final Month 24 study visit, participant weight will be tracked through study-provided BodyTrace smart scales. These scales use the cellular network to send participant weight data to BodyTrace servers, from which the investigators can directly download data. No identifiable information will be transmitted or stored on the BodyTrace servers; participants are identified only via a numeric study ID with no links to

personally-identifiable information. Frequency of self-weighing will be used in our *ADAPTIVE* risk algorithm to determine whether intervention should be triggered (see Figure 1 for details).

Data collected from FatSecret. Via the FatSecret API, we will download participant self-reported weight, physical activity, and dietary self-monitoring records (including food and drinks consumed along with portion size of these foods/drinks and information regarding kcal and macronutrient/micronutrient composition of these food/drinks). The FatSecret API does not provide timestamps of when participants self-monitor weight, caloric intake, or physical activity; however, our server will append time/day that data were downloaded to this dataset. MyTrack+ will sync with FatSecret in order to display summaries to participants (e.g., summaries of body weight, caloric intake, and physical activity over the past week). This data will be linked to participants only through a non-identifiable study identification number. Frequency of self-monitoring caloric intake will be used in our *ADAPTIVE* risk algorithm to determine whether intervention should be triggered (see Figure 1 for details).

Data collected from MyTrack+. Participants will be asked to respond to prompts to complete brief questionnaires via the MyTrack+ app. At the end of each week, participants will be prompted to respond to two questions in order to collect necessary data for the *ADAPTIVE* risk algorithm (see End of Week Questionnaire). Further, once each week (with participants unaware of the day that the questionnaire will be asked), participants will be asked to complete a 12-item questionnaire (see Weekly Questionnaire). Participants will also be able to launch FatSecret within the MyTrack+ app in order to enter weight, caloric intake, and physical activity. We will also collect usage data from this application in order to assess participant engagement. MyTrack+ will be connected to our research servers using only study identification numbers.

Statistical Design and Power

The primary outcome for the proposed trial will be weight change from Month 4 to Month 24. This variable will be continuous, and analyses conducted during our team's previous trials have demonstrated that weight change can be well modeled using a normal distribution.[3] Thus, standard parametric techniques will be used. A longitudinal mixed model will be used to analyze the primary aim data, using treatment condition and time since study start as fixed effects. One primary contrast of interest will be tested for significance: weight regain between Month 4 and Month 24 in the *ADAPTIVE* vs. *STATIC* treatment conditions. Fixed effects will include randomization condition, time since study start, and initial intervention weight loss (as a binary variable representing whether initial weight loss was ≥10% or <10%, used to stratify randomization). Sensitivity analyses will be conducted to assess robustness of results under alternative assumptions related to missing data (including a missing not-at-random approach assuming that individuals who discontinue participation in the study prior to Month 24 regained an average of .3 kg/month, up to baseline weight[3,56,57]).

The secondary outcome for the proposed trial will be the proportion of individuals in each group maintaining a clinically-significant (≥ 5% weight loss from baseline/Month 0)[4] at Month 24. Percent change will be calculated from assessment weights at Month 0 and Month 24, and participants will be categorized dichotomously as having weight losses at Month 24 of ≥ 5% or < 5% from baseline. Difference in proportion of participants maintaining a ≥ 5% weight loss will be assessed using a two-tailed z-test for independent proportions.

As an exploratory aim, we intend to compare weight loss trajectory over time between the two groups. For this, we propose to use longitudinal mixed-effects modeling[58–60] and the growth curve modeling[61,62] to analyze weight change data observed at several different time points, where the within-subject data correlated is accommodated by the random-effects terms included in the longitudinal mixed-effects model and by a general covariance matrix in the growth curve model. These analyses will demonstrate differences in weight change between participants in the *ADAPTIVE* and *STATIC* groups over time. Changes in dietary intake, physical activity, and self-monitoring adherence over time will be assessed using multilevel models for repeated measurements.[59] Potential treatment meditators (e.g., intervention engagement, operationalized as the proportion of possible extended-care sessions attended, adherence to self-monitoring, and changes in dietary intake/physical activity) will be assessed using bootstrapping to estimate confidence intervals around indirect effects.[63,64] Finally, the rich dataset collected via MyTrack+

(including time-stamped self-monitoring data (including not only frequency counts of self-monitoring behavior but also daily weight, caloric intake, and physical activity data) and ecological momentary assessment questionnaire data collected over a full two year study period) will be used to examine proximal predictors of change in weight and weight-related behaviors. Following the pragmatic framework developed by Nahum-Shani and colleagues,[65] we propose to build dynamic models of weight loss and weight regain, identifying key tailoring variables and potential intervention options for future just-in-time adaptive interventions for weight loss maintenance.

The most recent clinical guidelines for the treatment of obesity (released as a joint report by the American College of Cardiology [ACC], American Heart Association [AHA] and The Obesity Society [TOS])[10] define 2.5 kg as the *minimal* amount of weight loss associated with lowered risk of type 2 diabetes, and further note that weight losses of this magnitude are associated with modest improvements in triglycerides, and modest reductions in blood pressure. Thus, similar to previous weight management trials powered to detect this smallest clinically-meaningful difference,[3,28] sample size for the primary aim analysis was selected to provide a statistical power of .90 to detect a difference in weight change of 2.5 ± 5.5 kg between groups from Month 4 to Month 24. This resulted in an estimated 206 participants (103 participants per group). With SDs of 5.0 and 6.0, estimated power would be .946 and .845, respectively. Assuming a retention rate of 80% at Month 24, 258 participants would need to be randomized at Month 4.

Based on our previous studies,[3,20,28] we conservatively estimate that 60% of participants who complete the initial 16-week phone-based behavioral weight loss intervention will be eligible for randomization and ultimately randomized; thus, we propose to recruit 430 adults to take part in the initial weight loss program. Note that this sample size calculation is based on the planned contrast between groups at Month 24. With the observed data collected at the intermediate assessment times before Month 24, the calculated sample size should guarantee enough power for the longitudinal data analyses that include all observed data at different assessment times.

For the secondary aim, we propose to investigate the proportion of participants in each group who achieve a clinically-significant weight loss/maintenance defined by the more conservative Institute of Medicine[4] threshold (≥ 5% from baseline weight). The current ACC/AHA/TOS Clinical Guidelines for the Treatment of Obesity[10] note that maintenance of weight losses of ≥ 5% of baseline weight is associated with not only lower risk of developing type 2 diabetes, but also reductions in HbA1c of 0.6% to 1.0%, reductions in need for diabetes medications, clinically meaningful reductions in fasting glucose, improvements in LDL and HDL cholesterol, reduction in use of lipid-lowering medications, and reductions in systolic and diastolic blood pressure of 3 and 2 mm Hg, respectively. Further, using this cut-off will allow us to compare our results to previous weight loss maintenance trials. Previous trials of phone-based extended-care interventions demonstrate that between 38% and 44% of participants maintain weight losses of ≥ 5% between Months 18 and 24.[2,3,66,67] In assessing sample size needed and power for secondary aim analyses, we assume conservatively that 45% of *STATIC* participants will maintain clinically-significant[4] weight losses of ≥ 5% at Month 24. Under an intent-to-treat approach, including all randomized participants, we will assume any participant not returning for the Month 24 follow-up visit has *not* met this-cut off. With these assumptions, we assessed necessary sample size to power a 15%, 20%, and 25% difference between achievement of this clinically-significant cut-off in the *ADAPTIVE* and *STATIC* conditions. With a sample of 258 randomized participants (the estimated sample size needed to adequately power primary aim analyses), estimated power would be .676 for a 15% difference between ADAPTIVE and STATIC participants (i.e., with 60% and 45% of participants maintaining this clinically-significant cut-off, respectively); power for a 20% difference would be .902 and power for a 25% difference would be .985.

Data and Safety Monitoring Plan

Given that participants are not masked to intervention condition, vulnerable populations will not be enrolled, and this is a Phase II trial with minimal risks, we propose that the safety monitoring of the study will be conducted by Dr. David Janicke, the study Safety Officer. Dr. Janicke is a clinical psychologist and a Professor and Associate Department Chair in the Department of Clinical & Health Psychology at the University of Florida. His previous experience as principal investigator or co-

investigator on over 15 clinical trials (many of which focused on behavioral approaches to improving diet and physical activity) renders him well qualified to review aspects of the progress and safety of this trial. Information regarding progress with recruitment, retention at assessment sessions, reasons for dropout, and adverse events will be submitted once per year to the Safety Officer for review. After reviewing this data, the Safety Officer will indicate in writing to Dr. Ross and NIH whether the study is progressing appropriately or changes are needed. At any point during the trial, the Safety Officer can recommend that a phone call be held to discuss any issues with the progress or safety of the trial.

All of the staff involved in data collection will be trained by the Principal Investigator (Dr. Ross) and/or the Research Coordinator and must demonstrate competence in administering all measures. Study Research Assistants (masked to treatment condition) will review all assessment data for accuracy and completion, and the Lab Manager along with the Graduate Assistant in Biostatistics/Data Manager will monitor data entered into the REDCap system by participant self-report or by research staff and completion of ASA24[43] recalls via the National Cancer Institute online ASA24 portal. Participants will be re-contacted to provide missing data or to clarify ambiguous responses. Range checks will be built into the data entry procedure to alert staff to data that should be clarified. Under the supervision of the Research Coordinator and the Lab Manager, a complete double-entry verification procedure will be used for all data entered to ensure accuracy. Under the supervision of the Co-Investigator/study statistician (Dr. Qiu), the Graduate Assistant in Biostatistics/Data Manager will conduct error checking and preliminary analyses of all data to further ensure accuracy. Any existing hard copies of data will be stored in a locked filing cabinet in a locked room, and electronic data files will be password protected and backed up on a secure UF server.

All participant records and assessment data will be treated as confidential, including participants' names and the fact that they are participating in the study. Participant information collected by the research staff will contain only a non-identifiable study ID. A separate database linking study ID and participant identifiers (name, address, contact names and addresses) will be maintained in an encrypted, password-protected file stored on a secure UF server. Records and assessment data will be safeguarded according to the policy of UF's IRB, which promotes the protection of confidential health information.

All study workstations will be managed by UF Information Technology (IT), (workstations located in the Health Professions, Nursing, and Pharmacy building and the Dental Ground floor area of UF Health Shands Hospital will be managed by the College of Public Health and Health Professions IT, and workstations located in the Department of Computer and Information Science and Engineering (CISE) will be managed by CISE IT), patched regularly, will have lock screens that automatically engage at 15 minutes idle time. All study workstations will utilize GatorLink accounts for access, and study databases stored on these workstations will be encrypted and password protected. Only IRB-approved research team members will have access to study databases. Data will be encrypted during transfer from BodyTrace and FatSecret and when stored on UF servers. Participants will be assigned a unique, non-identifiable study identification number that will be used to link data from BodyTrace, FatSecret, MyTrack+, REDCap, and study databases.

Additional safeguards are in place to protect participant questionnaire data entered via REDCap. The REDCap platform is managed by the UF Clinical and Translational Science Institute and only IRB-approved research team members will have access to the platform. REDCap system security includes: secure logins, data encryption at rest, remote system logging and configuration and change management. Each team member is granted access to REDCap through a secure login, and audit trails are available by username. Data backups are encrypted both in flight and at rest.

7. Possible Discomforts and Risks:

a. Potential Risks

The extensive literature on behavioral interventions for weight loss shows that there are very few risks associated with this type of intervention, provided that energy intake exceeds 1,000 kcal/day.[68] In the proposed study, participants are encouraged to follow a balanced diet above this minimum amount (our targeted goals encourage participants to consume 1,200 to 1,800 kcal/day,

based on initial weight). This study asks some participants to monitor their weight daily; while this may raise concerns of negative psychological reactions (e.g., lower body image, depression), research has demonstrated that daily self-weighing does not negatively impact individuals.[69] There is a small potential risk of musculoskeletal injury associated with the exercise component of the intervention. In the Diabetes Prevention Program, on which the current initial intervention is based, the occurrence of musculoskeletal injuries was 3% higher in the lifestyle intervention group than in the placebo control condition.[70] In our studies, we have found that the risk of injury from exercise can be minimized through the use of moderate- rather than high-intensity exercise activities. The procedures to minimize risk are described in further detail below.

b. Protection Against Risks

The BMI cut-offs were chosen using current clinical guidelines, which recommend behavioral weight management intervention for adults with BMI ≥ 30 kg/m^2. Individuals with BMIs ≥ 45 kg/m^2 and individuals over the age of 70 years have greater medical comorbidities and require medical supervision during weight loss attempts; therefore, these individuals will be excluded from participation but will be provided with appropriate referrals. The remaining exclusion criteria are included for the safety of participants.

The behavioral weight management intervention will target a nutritionally-balanced energy intake that exceeds 1,000 kcal/day, thereby reducing the risks associated with a very-low-calorie diet.[10] Throughout the study period, participants will self-monitor dietary intake via the MyTrack+ app. During the initial 16-week intervention, study interventionists will review these records weekly, in consultation with the study team's Registered Dietitian (R.D.). Participants who are observed to be consuming nutritionally inappropriate intakes with regard to either the quantity or quality of foods consumed will receive individualized counseling to assist them in achieving appropriate intake. Any records that demonstrate food intake for < 800 kcal/day (the cut-point for "very low caloric intake," which typically requires medical monitoring[10]) for > 3 days in a week will be reviewed directly by the R.D., and an individual consult will be provided to the participant to assess risk. The R.D. will provide counseling as appropriate, and this additional intervention contact will be documented.

During the maintenance trial, it is likely that days with self-monitoring records demonstrating caloric intake totals of < 800 kcal represent days in which self-monitoring records were not fully completed. We counsel participants to record even just a meal or two if they cannot record the entire day. As a result, setting a rule to trigger nutritional counseling when MyTrack+ records demonstrate intake < 800 kcal/day is likely to lead to a large number of false triggers in both *ADAPTIVE* and *STATIC* maintenance programs. Therefore, during the maintenance trial, we propose to monitor this intake concurrently with weight change; similar to the algorithm we use to trigger additional intervention contact in the *ADAPTIVE* condition, we will program an automated monitoring system that will trigger a record review and consult by the R.D. if *both* of the following conditions are met: 1) a participant demonstrates a weight loss of ≥ 1.5% in a 7-day period, 2) a participant demonstrates > 3 days with caloric intake < 800 kcal/day. This safety monitoring will occur during the maintenance trial for participants in both the *ADAPTIVE* and *STATIC* conditions.

Prior to enrollment, participants who report diagnoses of type 2 diabetes, hypertension, or history of coronary heart disease will be asked to obtain written approval for participation from their physician, and any changes in medication required by reduction in caloric intake or weight will be managed by the participant's physician.

All written forms that contain identifying participant information will be stored in a locked, secure area, and only authorized personnel will have access to the records. Similarly, the computerized database housed in the University of Florida Clinical and Translational Science Institute (UF CTSI) will remain encrypted, and access will require password-protected authentication. Computer files will not include the participant's name; instead, a subject identification (ID) number will be used as a means of providing additional security. All audio recordings will be transferred from the recording device to UF servers immediately after each session, and the file deleted from the recorder. Audio recorders will be stored between uses in a locked cabinet in a locked office.

The current study collects data through a third-party smart scale (developed by BodyTrace, Inc.) and third-party smartphone app (developed by FatSecret). To protect participant privacy and confidentiality, no identifiable information will be provided to BodyTrace, Inc.; participants will be identified only by a study identification number. If social distancing procedures are in effect, participants will be notified in the informed consent document that their name and address will be provided to BodyTrace, Inc. for scale shipping purposes. Participants will be given the choice to set up their accounts with FatSecret using either their personal email address or a study-generated email address, and will be advised to refrain from sharing personally-identifiable information through the FatSecret application. Data from the FatSecret application will be downloaded to our servers linked with an assigned study identification number. Our study-developed smartphone application (MyTrack+) will further identify participants through a study identification number. Data will be encrypted during transfer between BodyTrace, Inc., FatSecret, and MyTrack+ and our UF-based research servers, and while stored on our servers. All computer workstations will utilize GatorLink accounts for access. All study databases will be encrypted and password-protected. Only IRB-approved research team members will have access to study databases.

8. Possible Benefits:

The potential benefits to participants include improvements in the quality of nutritional intake, increased physical activity, and weight loss. In individuals with obesity, such changes are often associated with improvements in health. Thus, the potential health benefits associated with participation appear to outweigh the small potential risk associated with lifestyle intervention.

At the end of each 2-year study period, participants in the maintenance program will be provided with a summary of their individual weight and data collected over the course of the maintenance program.

The development of successful approaches to the long-term management of obesity would represent an important scientific contribution and a benefit to society.

9. Conflict of Interest:

There are no conflicts of interest for any of the investigators.

10. Protocol Amendment History (all versions approved by the UF IRB)

Version	Date	Details
1	9/11/2019	1. Full protocol approved by UF IRB.
2	10/28/2019	1. Addition of two new measures (OPTION Scale and Body Image State Scale [BISS]) under “Assessment Schedule and Measures”.<br>2. Updated recruitment methods to include direct mail using publicly available mailing lists and advertisement of an IRB-approved flyer via Nextdoor.
3	03/20/2020	1. Updated “Initial Weight Loss Program” to note that group intervention sessions may be held by Zoom UFLPHI in the event of UF closure or emergencies.<br>2.Modification to “Assessment Schedule and Measures” to add “If social distancing procedures are in effect, weight will be measured using a study-provided BodyTrace smart scale.” [Following communication with our NIH PO, Month 4, Month 12, and Month 24 weights for Cohort 1 and all assessment point weights for Cohorts 2-5 were assessed via BodyTrace smart scale].
4	06/15/2020	1. Modification to “Orientation and final eligibility screening” to include conduct of orientation visits via Zoom and e-consenting via REDCap.

		2. Modification to “Screening Visit” to add procedure for mailing a study-provided scale to participants for weight to be measured.<br>3. Modification to “Assessment Schedule and Measures” to add the International Physical Activity Questionnaire (replacing use of ActiGraph monitors due to social distancing procedures)<br>4. Modification to “Assessment Schedule and Measures” to add “If social distancing procedures are in effect, a self-reported height will be recorded.”
5	03/16/2021	1. With COVID-19 restrictions still in place, in-person recruitment events are not possible. We have added remote-recruitment methods including the use of Facebook posts through local businesses, organizations and churches using materials previously-approved via the IRB. We will contact local businesses, organizations, and churches remotely (i.e., via phone and/or e-mail) to share IRB-approved recruitment materials. All inquiries will be directed to our lab's phone number or email so that our study team can respond.<br>2. Protocol update to remove pacemaker use as exclusion criterion. The scale that was originally planned to be used at initial grant submission had this exclusion; the scale that we have used since the original IRB approval (BodyTrace, Inc smart scale) does not use bioelectrical impedance and is safe to use for participants who can use cellular devices (a question has been added to the telephone screen to ask participants who have a pacemaker if they have any restrictions with cellular devices).
6	04/08/2022	1. Addition of one new measure (Self-Compassion Scale) under “Assessment Schedule and Measures”.
7	04/25/2023	1. Addition of one new measure (Program Satisfaction Questionnaire) under “Assessment Schedule and Measures”.
8	06/17/2024	1. Updated study team's titles and contact information<br>2. Modification to wording of exclusion criteria: “prior 6 months” changed to “6 months prior to screening)”.

REFERENCES

1. MacLean PS, Wing RR, Davidson T, Epstein L, Goodpaster B, Hall KD, et al. NIH working group report: Innovative research to improve maintenance of weight loss. Obesity. 2014;23(1):7–15.

2. Svetkey LP, Stevens VJ, Brantley PJ, Appel LJ, Hollis JF, Loria CM, et al. Comparison of strategies for sustaining weight loss: the weight loss maintenance randomized controlled trial. JAMA. 2008 Mar 12;299(10):1139–48.

3. Perri MG, Limacher MC, Durning PE, Janicke DM, Lutes LD, Bobroff LB, et al. Extended-care programs for weight management in rural communities: The Treatment of Obesity in Underserved Rural Settings (TOURS) randomized trial. Arch Intern Med. 2008 Nov 24;168(21):2347–54.

4. Institute of Medicine. Weighing the options: Criteria for evaluating weight-management programs. Washington, D.C.: National Academies Press; 1995.

5. Butryn ML, Webb V, Wadden TA. Behavioral treatment of obesity. Psychiatr Clin North Am. 2011 Dec;34(4):841–59.

6. Jeffery RW, Epstein LH, Wilson GT, Drewnowski A, Stunkard AJ, Wing RR. Long-term maintenance of weight loss: Current status. Health Psychol. 2000;19:5–16.

7. Klem ML, Wing RR, McGuire MT, Seagle H, Hill JO. A descriptive study of individuals successful at long-term maintenance of substantial weight loss. Am J Clin Nutr. 1997;66:239–46.

8. Perri MG, Corsica JA. Improving the maintenance of weight lost in behavioral treatment of obesity. In: Wadden TA, Stunkard AJ, editors. Handbook of Obesity Treatment. New York, NY: Guilford Press; 2002. p. 357–79.

9. Hall KD, Sacks G, Chandramohan D, Chow CC, Wang YC, Gortmaker SL, et al. Quantification of the effect of energy imbalance on bodyweight. The Lancet. 2011;378:826–37.

10. Jensen MD, Ryan DH, Apovian CM, Ard JD, Comuzzie AG, Donato KA, et al. 2013 AHA/ACC/TOS guideline for the management of overweight and obesity in adults. Circulation. 2014;129(suppl 2):S139-140.

11. Latner JD, Stunkard AJ, Wilson GT, Jackson ML, Zelitch DS, Labouvie E. Effective long-term treatment of obesity: a continuing care model. Int J Obes. 2000;24:893–8.

12. Ross Middleton KM, Patidar SM, Perri MG. The impact of extended care on the long-term maintenance of weight loss: a systematic review and meta-analysis. Obes Rev. 2012;13(6):509–17.

13. Dombrowski SU, Knittle K, Avenell A, Araújo-Soares V, Sniehotta FF. Long term maintenance of weight loss with non-surgical interventions in obese adults: systematic review and meta-analyses of randomised controlled trials. The BMJ [Internet]. 2014 May 14 [cited 2018 Jan 15];348. Available from: https://www.ncbi.nlm.nih.gov/pmc/articles/PMC4020585/

14. Wadden TA, Volger S, Sarwer DB, Vetter ML, Tsai AG, Berkowitz RI, et al. A two-year randomized trial of obesity treatment in primary care practice. N Engl J Med. 2011;365(21):1969–79.

15. Dutton GR, Gowey MA, Tan F, Zhou D, Ard J, Perri MG, et al. Comparison of an alternative schedule of extended care contacts to a self-directed control: a randomized trial of weight loss maintenance. Int J Behav Nutr Phys Act. 2017 Aug 15;14:107.

16. Grossi E, Grave RD, Mannucci E, Molinari E, Compare A, Cuzzolaro M, et al. Complexity of attrition in the treatment of obesity: clues from a structured telephone interview. Int J Obes. 2006 Jul;30(7):1132.

17. Dalle Grave R, Suppini A, Calugi S, Marchesini G. Factors associated with attrition in weight loss programs. Int J Behav Consult Ther. 2006;2(3):341.

18. Dalle Grave R, Melchionda N, Calugi S, Centis E, Tufano A, Fatati G, et al. Continuous care in the treatment of obesity: an observational multicentre study. J Intern Med. 2005 Sep 1;258(3):265–73.

19. Ross KM, Qiu P, You L, Wing RR. Characterizing the pattern of weight loss and regain in adults enrolled in a 12-week Internet-based weight management program. Obesity. 2018;26(2):318–23.

20. Ross KM, Wing RR. Implementation of an Internet weight loss program in a worksite setting. J Obes. 2016;2016(2016):9372515.

21. Breiman L, Friedman J, Stone CJ, Olshen RA. Classification and Regression Trees. Boca Raton, FL: Chapman and Hall/CRC, Taylor & Francis Group; 1984.

22. Hastie T, Tibshirani R, Friedman J. The Elements of Statistical Learning. New York, NY: Springer; 2009.

23. R Core Team. R: A language and environment for statistical computing. [Internet]. Vienna, Austria: R Foundation for Statistical Computing; 2022. Available from: http://www.R-project.org/

24. Therneau T, Atkinson B, Ripley B. rpart: Recursive Partitioning and Regression Trees. 2018.

25. Nahum-Shani I, Smith SN, Tewari A, Witkiewitz K, Collins LM, Spring B, et al. Just-in-time adaptive interventions (JITAIs): An organizing framework for ongoing health behavior support. University Park, PA: The Methodology Center, Penn State; 2014. Report No.: Technical Report No. 14-126.

26. Clinical and Translational Science Institute, University of Florida. Integrated Data Repository [Internet]. 2018 [cited 2018 Jan 14]. Available from: http://idr.ufhealth.org/

27. Wing RR. Behavioral approaches to the treatment of obesity. In: Bray GA, Bouchard C, editors. Handbook of Obesity: Clinical Applications. 2nd ed. New York: Marcel Dekker, Inc.; 2004. p. 147–67.

28. Perri MG, Limacher MC, von Castel-Roberts K, Daniels MJ, Durning PE, Janicke DM, et al. Comparative effectiveness of three doses of weight loss counseling: Two-year findings from the Rural LITE Trial. Obesity. 2014;22(11):2293–300.

29. Diabetes Prevention Program (DPP) Research Group. The Diabetes Prevention Program (DPP): Description of lifestyle intervention. Diabetes Care. 2002;25(12):2165–71.

30. Tudor-Locke C, Bassett DR. How many steps/day are enough? Preliminary pedometer indices for public health. Sports Med Auckl NZ. 2004;34(1):1–8.

31. Donnelly JE, Blair SN, Jakicic JM, Manore MM, Rankin JW, Smith BK. American College of Sports Medicine position stand. Appropriate physical activity intervention strategies for weight loss and prevention of weight regain for adults. Med Sci Sports Exerc. 2009;41:459–71.

32. Unick JL, Hogan PE, Neiberg RH, Cheskin LJ, Dutton GR, Evans-Hudnall G, et al. Evaluation of early weight loss thresholds for identifying nonresponders to an intensive lifestyle intervention. Obesity. 2014 Jul 1;22(7):1608–16.

33. Nackers LM, Ross KM, Perri MG. The association between rate of initial weight loss and long-term success in obesity treatment: Does slow and steady win the race? Int J Behav Med. 2010 Sep 1;17(3):161–7.

34. Ross KM, Wing RR. Impact of newer self-monitoring technology and brief phone-based intervention on weight loss: a randomized pilot study. Obesity. 2016;24(8):1653–9.

35. D'Zurilla TJ, Goldfried MR. Problem solving and behavior modification. J Abnorm Psychol. 1971;78(1):107–26.

36. Bandura A. Social cognitive theory of self-regulation. Organ Behav Hum Decis Process. 1991 Dec;50(2):248–87.

37. Bandura A. Health promotion from the perspective of social cognitive theory. Psychol Health. 1998 Jul 1;13(4):623–49.

38. Cullen KW, Baranowski T, Smith SP. Using goal setting as a strategy for dietary behavior change. J Am Diet Assoc. 2001 May;101(5):562–6.

39. Doran GT. There's a S.M.A.R.T. way to write management's goals and objectives. Manage Rev. 1984;70:35–6.

40. Ross KM, You L, Qiu P, Shankar MN, Swanson TN, Ruiz J, et al. Predicting high-risk periods for weight regain following initial weight loss. Obesity. 2024;32(1):41–9.

41. Ross KM, Shankar MN, Qiu P, Tian Z, Swanson TN, Shetty A, et al. Design of Project STAR: A randomized controlled trial evaluating the impact of an adaptive intervention on long-term weight-loss maintenance. Contemp Clin Trials. 2024 Nov;146:107707.

42. Harris PA, Taylor R, Thielke R, Payne J, Gonzalez N, Conde JG. Research Electronic Data Capture (REDCap) - A metadata-driven methodology and workflow process for providing translational research informatics support. J Biomed Inform. 2009;42(2):377–81.

43. Automated Self-Administered 24-Hour (ASA24®) Dietary Assessment Tool [Internet]. [cited 2018 Aug 3]. Available from: https://epi.grants.cancer.gov/asa24/

44. Ames GE, Heckman MG, Grothe KB, Clark MM. Eating self-efficacy: Development of a short-form WEL. Eat Behav. 2012 Dec 1;13(4):375–8.

45. Pinto AM, Fava JL, Raynor HA, LaRose JG, Wing RR. Development and validation of the weight control strategies scale. Obesity. 2013 Dec;21(12):2429–36.

46. Gibbs BB, King WC, Davis KK, Rickman AD, Rogers RJ, Wahed A, et al. Objective vs. Self-report Sedentary Behavior in Overweight and Obese Young Adults. J Phys Act Health. 2015 Dec;12(12):1551–7.

47. Roenneberg T, Keller LK, Fischer D, Matera JL, Vetter C, Winnebeck EC. Human activity and rest in situ. Sehgal A, editor. Methods Enzymol. 2015 Jan 1;552:257–83.

48. Leahey TM, Doyle CY, Xu X, Bihuniak J, Wing RR. Social networks and social norms are associated with obesity treatment outcomes. Obesity. 2015;23:1550–4.

49. Craig CL, Marshall AL, Sjostrom M, Bauman AE, Booth ML, Ainsworth BE, et al. International Physical Activity Questionnaire: 12-Country Reliability and Validity: Med Sci Sports Exerc. 2003 Aug;35(8):1381–95.

50. Sallis JF, Grossman RM, Pinski RB, Patterson TL, Nader PR. The development of scales to measure social support for diet and exercise behaviors. Preventive Medicine. 1987;16:825–36.

51. Cohen S, Kamarck T, Mermelstein R. A global measure of perceived stress. J Health Soc Behav. 1983;24:385–96.

52. Boggiano MM. Palatable Eating Motives Scale in a college population: Distribution of scores and scores associated with greater BMI and binge-eating. Eating Behaviors. 2016;21:95–8.

53. Herdman M, Gudex C, Lloyd A, Janssen M, Kind P, Parkin D, et al. Development and preliminary testing of the new five-level version of EQ-5D (EQ-5D-5L). Qual Life Res. 2011;20(10):1727–36.

54. Figueroa R, Saltzman J, Aftosmes-Tobio A, Davison K. Testing the construct validity of a brief measure of food, physical activity and sleep parenting in English and Spanish. Age. 2019 Mar;1(33):7–47.

55. Cash T, Fleming E, Alindogan J, Steadman L, Whitehead A. Beyond body image as a trait: The development and validation of the Body Image States Scale. Eat Disord. 2002 Jun;1(10 (2)):102–13.

56. Wing RR, Tate DF, Gorin AA, Raynor HA, Fava JL. A self-regulation program for maintenance of weight loss. N Engl J Med. 2006;355:1563–71.

57. Perri MG, Shankar MN, Daniels MJ, Durning PE, Ross KM, Limacher MC, et al. Effect of telehealth extended care for maintenance of weight loss in rural US communities: A randomized clinical trial. JAMA Netw Open. 2020 Jun 15;3(6):e206764.

58. Ten TH, Kunselman AR, Pulkstenis EP, Landis JR. Mixed effects logistic regression models for longitudinal binary response data with informative drop-out. Biometrics. 1998 Mar;54(1):367–83.

59. Fitzmaurice GM, Lipsitz SR, Ibrahim JG, Gelber R, Lipshultz S. Estimation in regression models for longitudinal binary data with outcome-dependent follow-up. Biostat Oxf Engl. 2006;7:469–85.

60. Qiu P, Zou C. Control chart for monitoring nonparametric profiles with arbitrary design. Stat Sin. 2010;20(4):1655–82.

61. Meredith W, Tisak J. Latent curve analysis. Psychometrika. 1990 Mar 1;55(1):107–22.

62. Panik MJ. Growth Curve Modeling: Theory and Applications. New York: John Wiley & Sons; 2014.

63. Bollen K, Stine R. Direct and indirect effects: Classical and bootstrap estimates of variability. Sociol Methodol. 1990;20:115–40.

64. Shrout PE, Bolger N. Mediation in experimental and nonexperimental studies: New procedures and recommendations. Psychol Methods. 2002 Dec;7(4):422–45.

65. Nahum-Shani I, Hekler EB, Spruijt-Metz D. Building health behavior models to guide the development of just-in-time adaptive interventions: A pragmatic framework. Health Psychol. 2015;34(Suppl):1209–19.

66. Sherwood NE, Crain AL, Martinson BC, Anderson CP, Hayes MG, Anderson JD, et al. Enhancing long-term weight loss maintenance: 2 year results from the Keep It Off randomized controlled trial. Prev Med. 2013 Mar 1;56(3):171–7.

67. Appel LJ, Clark JM, Yeh HC, Wang NY, Coughlin JW, Daumit G, et al. Comparative effectiveness of weight-loss interventions in clinical practice. N Engl J Med. 2011 Nov 24;365(21):1959–68.

68. National Heart, Lung, and Blood Institute. Clinical Guidelines on the Identification, Evaluation, and Treatment of Overweight and Obesity in Adults: The Evidence Report. Washington, D.C.: National Institutes of Health; 1998. Report No.: No. 98-4083.

69. Wing RR, Tate DF, Gorin AA, Raynor HA, Fava JL, Machan J. “STOP regain”: Are there negative effects of daily weighing? J Consult Clin Psychol. 2007;75(4):652–6.

70. Knowler W, Barrett-Connor E, Fowler S, Hamman R, Lachin J, Walker E, et al. Reduction in the incidence of type 2 diabetes with lifestyle intervention or Metformin. N Engl J Med. 2002;346(6):393–403.
