# Supplementary material for: Adaptive vs Monthly Support for Weight-Loss Maintenance: A Randomized Clinical Trial
Source: JAMA Netw Open. 2025 Sep 22;8(9):e2532681. doi: 10.1001/jamanetworkopen.2025.32681 (PMC12455375; doi:10.1001/jamanetworkopen.2025.32681)
Supplement: Supplement 2. — Data Sharing Statement [file jamanetwopen-e2532681-s002.pdf]

Data Sharing Statement

Ross. Adaptive vs Monthly Support for Weight-Loss Maintenance. *JAMA Netw Open*. Published September 22, 2025. doi:10.1001/jamanetworkopen.2025.32681

Data
Additional Information: Clinicaltrials.gov: NCT04116853
Data available: Yes
Data types: Deidentified participant data, Data dictionary
How to access data: Data can be requested from Kathryn M. Ross at Kathryn.Ross@aah.org
When available: With publication

Supporting Documents
Document types: Informed consent form
How to access documents: https://cdn.clinicaltrials.gov/large-docs/53/NCT04116853/ICF_001.pdf
When available: With publication

Additional Information
Who can access the data: Researchers whose proposed use of the data has been approved.
Types of analyses: Systematic reviews and meta-analyses
Mechanisms of data availability: Data will be made available with investigator support after approval of a proposal and signed data access agreement.
